# Reaching target LDL cholesterol has become more affordable with launch of ezetimibe/simvastatin combination in South Africa

**Published:** 2010

## Introduction

An affordable addition to the clinician’s choice of LDL cholesterol-lowering agents has become available in South Africa. Ashley Pearce, CEO of Merck Sharpe & Dohme, South Africa, and president of the Pharmaceutical Industry Association of South Africa (PIASA) emphasised the need to increase accessibility to innovative medicines for chronic diseases in South Africa.

Speaking at the launch of INEGY, Mr Pearce said, ‘After four to five years of deliberations within MSD, a very competitively priced combination tablet of ezetimibe (10 mg) with simvastatin has been launched in South Africa. We hope that clinicians and funders will make use of this opportunity to reduce cholesterol levels to the internationally recommended lower levels, particularly for high-risk patients where LDL cholesterol levels below 2.4 mmol/l are appropriate.’

Dr Dirk Blom, senior specialist at the Groote Schuur Hospital Lipid Clinic confirmed the value of this combination therapy in daily clinical practice at a meeting of clinicians held in Cape Town recently. Early clinical trials using statins for both primary and secondary prevention of coronary artery disease (CAD) have shown that for every 1-mmol/l reduction in lipid levels, there is a corresponding 21 to 24% reduction in coronary artery disease and vascular events.

‘Initial trials lowered LDL cholesterol by approximately 30% but significant residual risk remained. One of the approaches to lowering residual risk has been more aggressive LDL cholesterol reduction. The recent JUPITER study1 of rosuvastatin in primary prevention in patients with an LDL cholesterol below 3.6 mmol/l and raised hs-CRP levels showed that LDL cholesterol could be lowered to mean levels of 1.42 mmol/l safely and with benefit’, Dr Blom pointed out.

‘The combination of a statin and ezetimibe in a single tablet potently reduces LDL cholesterol via the dual mechanisms of reduced cholesterol synthesis and absorption. This combination can be very useful for patients who tolerate statins poorly, with statin-related myalgia being the commonest dose-limiting side effect. Ezetimibe is also very useful if the baseline LDL cholesterol is very high or when a very low LDL cholesterol target has been set.’

‘Doubling the statin dose only results in a further 6% reduction in LDL cholesterol levels, and reaching LDL cholesterol targets often requires multiple doublings which not all patients are able to tolerate. In this situation ezetimibe is a very useful add-on medication to background statin therapy, as adding ezetimibe further lowers LDL cholesterol to approximately the same degree as three doublings of the statin dose, without significantly increasing side effects’, he said.

‘The EASE study2 where ezetimibe was added to existing statin therapy showed a further 20 to 25% reduction in LDL cholesterol levels. The combination of ezetimibe and the statin was well tolerated. Ezetimibe’s action is mainly on the LDL cholesterol, although it does increase HDL cholesterol marginally and lowers triglycerides modestly.’

In addressing residual risk of patients on statin therapy, Dr Blom indicated that clinicians would need to be smarter at addressing the different components of the lipid profile, such as HDL cholesterol levels and increased triglycerides. ‘With other newer agents soon to come on the market in South Africa, clinicians will have a wider compendium of agents to choose from. While the evidence base for cardiovascular protection is strongest for statins, the complementary evidence is substantial for cardiovascular event reduction from LDL cholesterol lowering, generally using agents other than statins’, Dr Blom noted.

‘Despite the negative results of lipid lowering with added ezetimibe on the progression of aortic stenosis in the SEAS study, and the better effect of niacin on carotid intima–media thickness compared to ezetimibe in the ARBITER-6 study’, Dr Blom commented, ‘I feel confident to use ezetimibe when additional lowering of LDL cholesterol is required. However, the outcome study of ezetimibe therapy (IMPROVE-IT) is eagerly awaited. This study will provide data on the safety and efficacy of even lower LDL cholesterol targets and will finally answer the question whether lowering LDL cholesterol with ezetimibe improves outcomes to the same extent as seen with other LDL-lowering therapies, especially statins.’

‘The recruiting of the 18 000 patients needed for this study has recently been completed and results should be available in 2013, as the protocol requires a twoyear treatment period. The SEAS study also reported higher cancer rates in the ezetimibe arm, but analysis of much larger datasets from ongoing trials indicated that the findings in SEAS were likely due to chance and not a true drug-induced effect. This conclusion is supported by extensive pre-clinical data in which no carcinogenicity signal was observed.’

The ezetimibe/simvastatin combination has been flat priced at the different simvastatin levels. The combination is indicated as adjunctive therapy to diet for the reduction of elevated total cholesterol (total C), low-density lipoprotein cholesterol (LDL-C), apolipoprotein B (Apo B), triglycerides (TG) and non-high-density lipoprotein cholesterol (non-HDL-C), and to moderately increase high-density lipoprotein cholesterol (HDL-C) in patients with primary heterozygous familial and non-familial hypercholesterolaemia or mixed hyperlipidaemia. It is also indicated for the reduction of elevated total C and LDL-C levels in patients with homozygous familial hypercholesterolaemia (HoFH).

## Dosage and directions for use

The patient should be placed on a standard cholesterol-lowering diet before receiving the combination and should continue on this diet during treatment. The dosage should be individualised according to the baseline LDL-C level, the recommended goal of therapy, and the patient’s response. The tablet should be taken as a single daily dose in the evening, with or without food.

The dosage range is 10/10 mg/day to 10/80 mg/day. The recommended usual starting dose is 10/20 mg/day. Initiation of therapy with 10/10 mg/day may be considered for patients requiring less aggressive LDL-C reductions. Patients who require larger reductions in LDL-C (> 55%) may be started at 10/40 mg/day. After initiation or titration of the combination, lipid levels may be analysed after two weeks and dosage adjusted, if needed.
